# Three Cases in Oral Surgery1Read before the American Academy of Dental Science, Boston, February 1, 1905.

**Published:** 1905-11

**Authors:** Thomas Fillebrown

**Affiliations:** Boston, Mass.


### THREE CASES IN ORAL SURGERY.[1]

BY THOMAS FILLEBROWN, M.D., D.M.D., BOSTON, MASS.

I HAVE had in my practice the three cases which I have contemplated for some time bringing to the attention of the Academy. The first one I mention particularly to illustrate what the X-rays might have done. Some years ago, before Dr. Clapp had his X-ray machine, and before the value of X-ray examinations was realized, a young woman about twenty-eight years old came to me, showing a trouble in the region of the left inferior third molar, evidently caused by a tooth. She had had an attempt made once or twice to get the tooth out, but the operators had been unable to reach it. It was then discharging continually. I probed the matter through the fistula, and it was plain that there was some part of a tooth there, but it was so low down that a considerably serious operation was demanded to reach it. I took her to a hospital, where she could remain, and with a trephine in the dental engine I cut down the lingual wall of the alveolus of the jaw to, as I thought, about the level of the apex of the root of the second molar. I carried my trephine down, thinking that

---

[1] Read before the American Academy of Dental Science, Boston, February 1, 1905.

I might bore out the substance of the tooth that was remaining. The jaw-bone was so dense that I could not in that case determine, as I usually can, the line of demarcation between the root and the bone. There is usually to my touch a difference in feeling sufficient for me to recognize bone from tooth. I got down until I thought I had all of it out.

The lady had to go away before it had healed. Her duties called her to Europe, and the jaw set seemingly well. She later went South, I think to Washington, and as it then gave her some trouble, a surgeon operated on it, and attempted to remove the same. Some three or four years after I had first operated she drifted back to Boston, and came into my hands again. I probed around it, and plainly felt the outline of a root of a tooth. After all that time, and the many operations, the parts had greatly softened up and the tooth had risen a little, and I found a point that seemed to be favorable to getting hold of it. I took an elevator and, using it carefully, drew out the remains of a wisdom-tooth. Of course, the patient got well in a short time after that. The point I make in reporting this case is, that had we known of the X-ray and realized its value, and had I used it on this lady, I should have found an embedded wisdom-tooth and known how deeply it was buried. I think it was more deeply embedded than the famous case Dr. Cryer has figured in his work. There is another point to consider. Had it been diagnosed at the time, would it have been wiser to remove it at the time, or wait and temporize with it, as was done? The patient would have been saved much annoyance had it been done at once, though it would have required quite an extensive and severe operation.

The second case is quite peculiar. In February, 1903, a lady, aged about forty, married, came to my office, with partial anky-losis of the jaw. She could open her teeth but very little. Grad-ually the mobility had been lessened until at that time it was with difficulty that she could get food enough between her teeth to sustain life. There were two lower inferior molars, one on each side, so that from the bicuspids to the first molars there were no teeth. She had had trouble with them before. Both had been devitalized and were filled. I did not think it best at first to interfere with this. At that time, as now, I was using suggestion considerably, and I concluded to try its merits. Between then and April I saw her quite a number of times, and she got great relief, so that she regained half of the mobility which she had lost, and was able to feed herself fairly well. But there was considerable pain. I could not detect that there was anything abnormal about the articulation of the jaw, although she suffered much pain in that region, as it did not get well. In April I concluded that I would prove the quality of the operations. I removed the fillings and examined the pulp-chambers, and found in one of them very sensitive pulp in the root. I treated this awhile, removed the sensitiveness, and refilled the teeth. There was still more improvement, but not great. She did not get entirely well, and in May we finally concluded that she had better have the molars out. The desire to save them was for the purpose of using them later as abutments for bridge-work. Although improvement followed, she did not fully recover. Near the last of my treatment I learned that her husband was suffering from a trouble from the effects of which he died in June. The great mental strain the patient was under, and she being so reduced physically, retarded the cure. Of course, being relieved of all that care, responsibility, and worry, she improved in health, and as she improved in health the gain that she made in the mobility of the jaw was maintained and increased and the pains disappeared, and when I saw her last she was in very good condition. It was trying to me to take out two strong, firm teeth, but I could see no other way. There is some excementosis on the roots, but not enough to account for so much trouble.

Speaking of excementosis recalls a case I had thirty or more years ago, where an upper molar in a gentleman's mouth was causing him to suffer extreme pain. I removed the pulp, and tried all sorts of treatment, which failed to give any relief. By exclusion, I diagnosed excementosis. I extracted the tooth and found a large deposit. The patient was entirely relieved from pain. Within a year or two he had another tooth that acted just the same way, and, guided by my former experience, I extracted it, and found it in the same condition.

*Dr. Wilson.*—Did these two teeth you extracted have any cavities in them?

*Dr. Fillebrown.*—I do not remember that there were any cavities in them. The memory of the case is called up suddenly. I am not positive.

My third case is of a man about thirty, who called on me in

1902. Some four years before that he had had the right inferior first molar tooth extracted, a root being left. His face continued to give him much trouble, with some swelling for about two years. He had called once or twice on the dentist who had extracted the teeth, and did not get much satisfaction, and finally called on another and had the root out. Trouble continued. He consulted several other dentists, and got no relief. Finally, a lawsuit for damages was the result, and he lost his case. He had consulted so many dentists that the responsibility, if any, was too much divided. He came to me after the matter was quite far advanced, and I operated on it, hoping to stir up the matter so as to get some activity in the parts, but I did not succeed, although I tried three times. Finally, the trouble seemed to locate in the anterior portion of the diseased part, and a great deal of pus was continually welling up from the vicinity of the root of the second bicuspid. I examined his bicuspids carefully many times, and there was no visible sign of trouble with them, and it was impossible for me to believe that they were the source of any constantly discharging abscess. I made up my mind to sacrifice the bicuspids, and I extracted the teeth. I broke open the second bicuspid and found that the pulp was completely calcified, white in color. There is the root, and the pulp standing up intact, seemingly of perfectly natural shape. It was a surprise to me. I saw him later, and the abscess caused by the tooth was cured, but there was well-developed necrosis in the region of the first molar, the exfoliating process being extremely slow, it will be some time before he gets entire relief. I did not feel warranted in cutting to any great extent at that time.

DISCUSSION.

*Dr. Hopkins.*—I should like to ask Dr. Fillebrown, in regard to the first case, the wisdom-tooth, whether it would have been possible to have done anything by extraction of the second molar?

*Dr. Fillebrown.*—Possibly, if we had known what was there. It seemed hardly reasonable to do so at that time.

*Dr. Hopkins.*—I had such a case in the Massachusetts General Hospital last summer. I went down there on call by one of the surgeons. I had the assistance of the hospital. By getting out the second molar I was able to get out a wisdom-root that was difficult of access where there was partial ankylosis of the jaw.

*Dr. Fillebrown.*—That certainly was a good thing to do.

*Dr. Potter.*—I would like to report a case that came to me two or three years ago, a case of a young lady about eighteen or twenty years of age, who had a fistula through the face, about opposite the superior first molar. She had been in the hands of a well-known surgeon of this city for treatment. He was washing out the fistula and trying to heal it up. I could never find out just what his diagnosis of the trouble was. On examination of the left superior maxilla, I found a suspicious second bicuspid. The bicuspid had the history of being a tooth in which the pulp had died. The patient said that she had been treated. The root-canal in this tooth had been treated, so that one would naturally expect that the second bicuspid was the source of this trouble. That was the natural supposition. I percussed the first and second bicuspid, and the first molar. The only indication of trouble was in this second bicuspid. I naturally opened into the root of the second bicuspid, to see if there was any action between that and the fistula. After opening it and undertaking to force a stream of water through, I found there was no free opening. There was a large filling in the proximal surface of the first molar, and although this molar gave no indication of trouble, I thought I would open into it, which I did, and found a dead pulp, and it was not long before I had forced a stream of water through the first molar and out through the cheek. The case is singular, in that the first molar had never given the slightest symptom of trouble. The patient could remember nothing unfavorable from that first molar, and yet there had been sufficient trouble there, sufficient force and energy in the pathological process, to force the fistulous opening through the cheek. The case healed quickly with the renovation of the first molar, and the scar was extremely slight.

*Dr. Fillebrown.*—In a condition where a fistula appears in the region of a bicuspid on the cheek, is it not evident that the trouble is with the molars and not with the bicuspid?

*Dr. Andrews.*—I had a case brought to me by one of the physicians of Cambridge, a case of a fistula under the chin. Several dentists pronounced the teeth all right. It was curetted, but it did not heal. I examined the lower incisors carefully. There was no sensation on percussion. I thought I would use my electric light. I found one of the centrals much darker than the other. I drilled in and found a dead pulp. I brought that on in three weeks' time, and it commenced to heal, and healed up in good shape. So that we cannot always tell by the appearance of the teeth where there is a dead pulp.

*Dr. Potter.*—I would like to ask Dr. Fillebrown if he would not expect soreness and trouble from all teeth that had produced a fistula?

*Dr. Fillebrown.*—Yes; but there are exceptions enough to that to put one continually on his guard.

*Dr. Wilson.*—I think I can answer that question. I remember a good many years ago a young patient came to me with her mother, a young girl, I should think fifteen or sixteen years old, and I noticed a little bunch under her chin. I asked what that was, and she said, "I think it is a tumor, and Dr. —— is going to take it out, as it does not heal." The gentleman named was one of our most prominent surgeons in Boston. I did not say anything at the time, but I suspected it was a tooth. I made a very careful examination, and found it was the sixth-year molar tooth. I told the mother so at the time, and said, "I am quite sure that I can cure this, and there will be no need of an operation." She was quite sceptical at the time, because the man who was to perform the operation was one of the most skilful surgeons in Boston. To make a long story short, I probed the tooth, the fistula healed up, and she has never had any trouble since. There was no pain or soreness. The tumor dried up. It was a little cauliflower excrescence.

*Dr. Lett.*—I want to relate a peculiar case. A lady was sent to me when in her eighth month of pregnancy with a small tumor, the size of a large pea, right under the second bicuspid tooth. The bicuspids were perfect, without any fillings. The second and first molar had been extracted. On account of the closeness of the period of delivery, her physician advised that nothing be done then. Two months later I saw her, and the tumor had grown to the size of a pigeon's egg, and went down through to a thin layer of bone, in which there was an enormous pus socket, which looked like this Golden Flake sealing-wax. The bicuspid teeth were sensitive at the cervical margin, so I did not disturb them. This woman had fallen out of a third-story window when about two years old. It was a very interesting case for the surgeons of Boston, who did not see how she could live. She had a third molar tooth that had two small gold fillings and a vital nerve. The cervical margin was quite sensitive. I could get a probe as far as the mesial root of the second molar. I thought of extracting that tooth, but was very much in doubt about it, when the lady's relatives partially decided for me by demanding the extraction of the tooth. I did not see its use, so I decided to take it out, and found quite a severe necrotic condition. The apex of the two roots had been eaten away by this necrotic process. Immediately after this it started to heal, and accomplished this beautifully and entirely. I do not know why I took the tooth out; it was under the impulse of the moment. I do not see why that necrotic condition existed there and caused the condition so far anterior to it.

*Dr. Fillebrown.*—Dr. Lett's remark leads me to express a conviction that I have had for a good while, that where we have these serious troubles with the jaws, that cannot be controlled, it is advisable, and is demanded of the operator, that he make examinations, and seek for a cause which is oftentimes remote from the effect. I believe it to be our duty not to be too conservative in the matter of removing teeth, because health is worth more than any one tooth.

Many of you perhaps remember the article by Dr. Seton, of New York, who said that there was too much saving of teeth, speaking particularly in regard to troubles of the ear and the eye, and also making the statement that dentists were saving too many dead teeth. I remember that Dr. Abbott took umbrage at it, and wrote several severe articles in reply. I believe Dr. Seton was right. There is something besides teeth in the human system, and when teeth are less useful than the parts to which they are doing damage they ought to be removed.

*Dr. Potter.*—As an incident of office practice, I want to report the use of a very satisfactory instrument that one of our members has been working over for several years. You have heard Dr. Merriam speak with some feeling of the fact that he had introduced something here in the Society long ago, and no notice was ever taken of it. I think that when our members do anything in the way of processes or instruments, we ought to take some notice of it.

I have been using Dr. Wilson's plugger for only a short time, but it seems to me rather a valuable thing. If any of you have not been using it, I think you ought to try it. It seems to me that it manages the gold better than a spring mallet. It requires less muscular energy to work it. It is refreshing to get hold of a plugger that works so easily.

*Dr. Bradley.*—Speaking of Dr. Merriam's introduction of gutta-percha, I do not know that there is any formal record of his work in gutta-percha, but almost every one knows that he was one of the pioneers in the use of it. I remember twenty years ago when he demonstrated the use of gutta-percha and the oil of cajuput and taking impressions in the root-canals, and I know that he has visited Rhode Island and demonstrated the use of gutta-percha and talked about it there. He not only spoke about it and demonstrated its use, but left a box for every member that was present. I was looking over my things recently, and found some of his gutta-percha. I feel sure, Mr. President, that we want to give Dr. Merriam a great deal, if not all, of the credit for unfolding to us a more complete knowledge of the possibilities that are in gutta-percha.

*Dr. Fillebrown.*—I wanted earlier to say a word on that very point. I think Dr. Merriam is acknowledged as being the pioneer in the use of gutta-percha. I think it is on record sufficiently. He wrote a paper, and read it here and in other places. It has been in general use ever since that. I am sure that if you speak to the Harvard alumni, you will find very few of them ignorant of his use of gutta-percha. Dr. Hamilton did not claim to originate the use of gutta-percha. He was only using what others were using.

H. S. Parsons,
*Editor.*

